# 小细胞肺癌免疫治疗疗效预测指标的现状与未来

**DOI:** 10.3779/j.issn.1009-3419.2020.101.42

**Published:** 2020-10-20

**Authors:** 雅凝 杨, 燕 王

**Affiliations:** 100021 北京，国家癌症中心/国家肿瘤临床医学研究中心/中国医学科学院北京协和医学院肿瘤医院内科 Department of Medical Oncology, National Cancer Center/National Clinical Research Center for Cancer/Cancer Hospital, Chinese Academy of Medical Sciences and Peking Union Medical College, Beijing 100021, China

**Keywords:** 肺肿瘤, 程序性死亡受体-1, 程序性死亡配体-1, 疗效预测, Lung neoplasms, Programmed cell death 1, Programmed cell death-ligand 1, Efficacy predictors

## Abstract

近年来，免疫治疗在小细胞肺癌领域取得了令人瞩目的突破，为患者带来生存获益。然而，现有的临床研究结果表明试验组通常在治疗开始3个月-6个月以后方可看出获益的趋势，因此如何筛选优势人群是免疫治疗研究的重点。目前已有的临床试验对生物标记物进行了不断地探索，但结果不尽一致。我们亟需有力可靠的疗效预测指标有效地筛选优势人群、扩大受益群体。故本文将对小细胞肺癌免疫治疗疗效预测指标的现状及未来的发展前进方向展开阐述。

## Abstract (en)

Inhibitions of immune checkpoints, including programmed cell death 1-programmed cell death-ligand 1 (PD-1-PD-L1) axis, cytotoxic T lymphocyte associated antigen-4, are at the forefront of immunotherapy for small cell lung cancer. However, the survival benefits emerged after three to six months since therapy initiation. A growing body of evidence suggested that the current biomarkers were not consistent and convincing. Here, we review the current biomarkers including PD-L1, tumor mutation burden, DNA damage response pathway. Furthermore, we elucidate the potential biomarkers, such as tumor-infiltrating lymphocytes, circulating tumor cells.

国家癌症中心于2019年1月发布了最新的癌症统计数据报告，无论是发病率还是死亡率，肺癌稳居榜首。小细胞肺癌（small cell lung cancer, SCLC）是一种起源于支气管黏膜上皮的Kulchitsky细胞的异源性神经内分泌肿瘤，是肺癌中侵袭性最强的亚型，约占肺癌的15%-20%。70%以上患者发现时已是广泛期SCLC（extensive stage SCLC, ES-SCLC）^[1]^，既往其一线标准治疗为依托泊苷联合顺铂或卡铂^[2, 3]^，但此方案预后较差，中位生存时间仅为10个月，2年生存率不足5%^[4]^。随着免疫时代的到来，SCLC也迎来了治疗的变革。

程序性死亡受体-1（programmed cell death 1, PD-1）及程序性死亡配体-1（programmed cell death-ligand 1, PD-L1）抑制剂是目前的研究热点。PD-1和PD-L1结合可通过募集酪氨酸磷酸酶（SHP-1和SHP-2）抑制T细胞的信号转导，下调T细胞活性并诱导T细胞凋亡，从而起到免疫抑制作用^[5]^。因此有效地阻断PD-1/PD-L1可恢复T细胞功能，使抗肿瘤应答正常化。根据CheckMate032研究和KEYNOTE-028/158研究结果，Nivolumab（纳武利尤单抗）^[6]^和Pembrolizumab（帕博利珠单抗）^[7]^相继被美国食品药品监督管理局（Food and Drug Administration, FDA）批准用于ES-SCLC患者的二线及二线以上治疗。IMpower133及CASPIAN研究在ES-SCLC患者一线治疗中均取得了总生存期（overall survival, OS）的突破性进展，由此FDA批准PD-L1单克隆抗体Atezolizumab（阿特珠单抗）^[8]^及Durvalumab（德瓦鲁单抗）^[9]^与卡铂和依托泊苷联合用于ES-SCLC患者的一线治疗。但是，在2020年美国临床肿瘤学会（American Society of Clinical Oncology, ASCO）年会中，KEYNOTE-064研究没有取得令人满意的结果。尽管实验组的无进展生存期（progression-free survival, PFS）明显提高，但是OS没有得到明显改善。

无论免疫单药治疗还是免疫联合治疗，SCLC患者可从中得到不同程度的获益。但是免疫治疗仍然存在一定的局限性。接受免疫单药或免疫联合化疗组的患者通常是在治疗的3个月-6个月后才能从中明显获益，因此如何优化治疗人群，让患者更早地从免疫单药治疗或免疫联合治疗中获益以及如何寻找可靠的疗效预测指标成为精准治疗背景下亟待解决的问题。本文将主要对PD-1/PD-L1抑制剂治疗SCLC患者疗效预测的相关研究及未来潜在的发展方向进行综述。

## 1 基于肿瘤本身的检测

### 1.1 PD-L1的表达水平

PD-L1蛋白表达的高低对免疫治疗疗效的预测价值在非小细胞肺癌（non-small cell lung cancer, NSCLC）中已经得到了验证。CheckMate 057^[10]^、KEYNOTE-010^[11]^及POPLAR研究^[12]^的亚组分析均提示，高表达PD-L1的患者在免疫治疗中获益较多。但现有研究尚无法充分证明PD-L1蛋白表达的高低与SCLC免疫治疗疗效的相关性。KEYNOTE系列研究如KEYNOTE-028、KEYNOTE-158研究表明，根据PD-L1蛋白的表达水平情况，研究者可筛选出接受Pembrolizumab获益的SCLC患者^[7]^。但是其他的结果却与此不同。无论是IMpower133及CASPIAN研究结果，还是CheckMate032研究，均提示PD-L1表达水平与试验组疗效并无相关性^[6, 9, 13]^。以上不同的研究结果可能是由于SCLC患者的PD-L1表达较低（10%-40%）^[6, 14, 15]^，IMpower133研究及CASPIAN研究中PD-L1表达阳性（PD-L1 > 1%）的患者比例均在6%左右，所以研究结果的差异可能是因为较少的阳性样本量所致。今后的研究可能需要纳入更多的SCLC患者，进一步探索PD-L1表达水平与免疫疗效之间的相关性。

目前对于PD-L1的检测也有一定的争议。一方面，目前FDA批准用于检测PD-L1的抗体主要包括22C3、28-8、SP142及SP263，前两者对应Dako诊断平台而后两者则对应Ventana诊断平台，对于PD-L1阳性的截断值定义各不相同。近几年国际上已有不少研究评估不同抗体检测的一致性，2017年评估这4种抗体一致性的蓝印计划（The Blueprint Project）的Ⅰ期结果显示，22C3、28-8及SP263的检测结果较为相似，具有可交换性，而SP142检测的肿瘤细胞阳性百分比则较低^[16]^。另一方面，有研究^[17]^显示，对于有多个病灶的肺癌患者而言，不同病灶的PD-L1表达情况有所差异，换言之肿瘤的异质性决定了以少量细胞样本代表整体是有失偏颇的。综上，PD-L1仍有待进一步的研究评估，目前尚不足以成为SCLC可靠的预测指标。

### 1.2 肿瘤突变负荷（tumor mutation burden, TMB）

TMB即所评估基因的外显子编码区每兆碱基中发生替换、插入、缺失突变的数量。突变的产生有利于T细胞的识别，从而激活免疫应答，因此可在一定程度上反映疗效。该指标目前已被FDA批准可以用于泛瘤种免疫治疗的疗效预测指标。既往研究^[18]^表明，SCLC较NSCLC具有更高的TMB：平均约为7.37 mut/Mb。既往NSCLC研究中，无论是组织TMB（tissue-based TMB, tTMB）还是血液TMB（blood-based TMB, bTMB）在免疫单药疗效的预测价值较高，但是在免疫联合治疗的预测能力仍需进一步探讨。*Nature Medicine*上发表的一项验证性研究^[19]^将OAK研究及POPLAR研究的bTMB与tTMB进行配对比较，两者呈相关关系（rs=0.64, 95%CI: 0.56-0.71），敏感性为64%，特异性为88%。但是在SCLC的研究中，关于TMB的探索相对匮乏，且结果不尽一致。

在CheckMate032研究中，401例患者中有211例接受了组织的全外显子测序，并按TMB表达高低分为低表达（0 mut/Mb-143 mut/Mb）、中表达（143 mut/Mb-247 mut/Mb）及高表达（≥248 mut/Mb）三组，无论在Nivolumab单药组还是联合Ipilimumab治疗组，高TMB表达组均表现出更佳的结局：在客观缓解率（objective response rate, ORR）方面，单药治疗组中高、中、低三组分别为21.3%、6.8%及4.8%；在联合治疗中ORR则分别为46.2%、16.0%及22.2%；PFS和OS也得到了显著改善，尤其是在联合组中更为明显，OS达到了22个月^[20]^。然而在IMpower133研究中，TMB的疗效预测效果欠佳。IMpower133研究将bTMB作为疗效预测的生物标记物，将截断值定义为10 mut/Mb和16 mut/Mb，遗憾的是，分析显示bTMB与ORR、PFS、OS等无关^[8]^。尽管以上两项研究的TMB分别来源于组织和血液，但是结果差异更多可能是由于研究人群接受的治疗分别是免疫单药和免疫联合治疗。所以如何进一步优化TMB的算法，使其可以区分免疫联合治疗的优势人群是未来研究的重点。

近期有一篇研究^[21]^通过优化TMB的算法，进而探索低频TMB与免疫单药治疗的疗效预测相关性，研究结果显示低频TMB与免疫单药的疗效关系密切。同时，另一篇回顾性研究^[22]^提示低频TMB与化疗治疗的疗效也有相关性。尽管以上均是在NSCLC中进行的探索，目前尚无关于SCLC方面的研究结果，但是低频TMB与SCLC之间的相关性在未来的研究中可以进一步探索与分析。

**1:** PD-1/PD-L1抑制剂治疗SCLC相关临床试验中PD-L1情况 Summary of PD-L1 expression in clinical trials of SCLC patients received PD-1/PD-L1 inhibitors

	IMpower133	CASPIAN	CheckMate 032	KEYNOTE-028	KEYNOTE-158
Interpretation	Tumor cells and immune cells	Tumor cells and immune cells	Tumor cells	Tumor cells	Tumor cells
Definition of PD-L1 positive	TC/IC expression≥1%	TC/IC expression≥1%	TC expression≥1%	PD-L1 expression≥1%	PD-L1 expression≥1%
PD-L1 positive rate	TC≥1%: 6.2%	TC≥1%: 6%	18%	31.7%	39%
		IC≥1%: 50.4%	IC≥1%: 22.3%		
PD-L1 assays	Ventana SP142	Ventana SP263	Dako 28-8	Dako 22C3	Dako 22C3
Treatment line	First line	First line	≥Second line	≥Second line	≥Second line
TC: tumor cells; IC: immune cells; PD-1: programmed cell death 1; PD-L1: programmed cell death-ligand 1.					

### 1.3 *DDR*基因

DNA损伤应答（DNA damage response, DDR）通路的受损被认为是预测铂敏感性的生物标记物。2019年ASCO上发表的一项研究显示，具有*DDR*基因突变的晚期NSCLC患者具有更好的免疫疗效。此研究中共招募468例接受免疫治疗的患者，DDR阳性定义为具有致病性DDR改变的病例，共有74例。DDR阳性组的TMB中位值明显高于阴性组（12.1 mut/Mb *vs* 9.8 mut/Mb, *P*=0.007），且具有明显更高的ORR（31.1% *vs* 19.1%, *P*=0.03）和PFS（16.3个月*vs* 9.8个月，*P*=0.009）^[23]^。另外一项研究^[24]^分析了TCGA及ICGC数据库包括29个瘤种在内的*DDR*通路基因、TMB及新抗原数据，探究DDR与TMB及新抗原的关系，并纳入接受免疫治疗的NSCLC及黑色素瘤患者来验证DDR与临床疗效的关系，结果表明≥2条DDR通路的共突变是TMB的良好替代指标，并能在一定程度上反映临床疗效。近期韩国的一项研究对166例SCLC患者进行基因测序，发现SCLC的DDR通路改变与TMB相关，尤其是双链DNA断裂（double-strand breaks, DSB）和单链DNA断裂（single-strand breaks, SSB）与高TMB呈正相关^[25]^。*DDR*基因是另一新兴的潜在生物标记物，考虑其与TMB的高度相关性，能否在未来的临床应用中优化或替代TMB检测也有待进一步的考证。

### 1.4 其他突变基因情况

既往研究^[18, 26-28]^显示，SCLC中最常见的突变基因为*TP53*（77%-94%）、*RB1*（31%-79%），紧随其后的则为MYC家族、*SOX2*、*FGFR*的基因扩增，关于PI3K/AKT/mTOR通路的突变也并不罕见。上述基因在多个层面影响肿瘤细胞的增殖、侵袭、凋亡等多个过程，因此近年来有研究探索SCLC的突变基因与其预后的关系。美国一项纳入了50例一线接受化疗的SCLC患者的回顾性研究^[29]^显示，*RB1*基因突变患者拥有更好的生存获益，两组的mOS分别为11.7个月和9.1个月（*P*=0.04），mPFS分别为11.2个月和8.6个月（*P*=0.06）。而*MYC*突变、PI3K/AKT/mTOR通路的突变则与较差的生存预后相关^[30, 31]^。一项共纳入了220例接受化疗或放化疗的SCLC患者的回顾性研究^[32]^提示*TP53*基因突变阳性的局限期SCLC患者生存预后较差（mOS：15.3个月*vs* 20.1个月；mPFS：7.7个月*vs* 9.2个月），*PI3K*/*AKT*/*mTOR*基因突变也表现出了同样的趋势（mOS：4.6个月*vs* 9.4个月；mPFS：2.9个月*vs* 4.8个月）。现有的研究提示SCLC的基因突变对于化疗或放化疗疗效有一定的预测价值。但遗憾的是，目前尚无研究报道免疫治疗中疗效和突变基因的关系，期待未来的研究可以进一步地深入该领域。

## 2 肿瘤微环境的检测

### 2.1 肿瘤浸润淋巴细胞（tumor-infiltrating lymphocyte, TIL）

TIL是肿瘤免疫微环境中的重要组成部分，环境中TILs的主要组成部分是CD8^+^细胞毒性淋巴细胞、CD4^+^辅助性淋巴细胞及CD4^+^调节性淋巴细胞。2017年*Nature*上发表的一篇综述提出根据CD8^+^ T细胞将免疫表型分为免疫炎症型、免疫豁免型及免疫沙漠型三种^[33]^。在一项Durvalumab联合奥拉帕利治疗SCLC的Ⅱ期临床试验中，共纳入了14例可评估的患者，其中9例表现为免疫豁免型，3例表现为免疫炎症型，2例表现为炎症沙漠型。由此可见，SCLC以免疫豁免型为主，而免疫治疗药物更倾向于在CD8^+^ T细胞浸润的免疫微环境中发挥作用^[34, 35]^。几项回顾性研究均探索了CD8^+^ TIL密度与预后的关系：一项研究^[36]^共纳入了56例SCLC术后患者，其中42例呈CD8^+^ TIL高密度，结果分析显示高CD8^+^ TIL密度与总生存期呈正相关关系（HR=0.429, *P*=0.008）。另一项近期发表在*Journal of Thoracic Oncology*上的研究^[37]^则对比了23例总生存期 > 4年的SCLC患者和18例总生存期≤2年患者的TIL，结果同样显示CD8^+^ TIL密度是改善SCLC总生存期和无病生存期的独立预后因素。TIL的预测价值不仅在SCLC中崭露头角，在其他瘤种中也展现了一定的作用。Geng等^[38]^研究发现，对于接受Nnivolumab治疗的NSCLC患者，PD-1表达阴性的CD8^+^ TIL数量与免疫疗效呈正相关。在黑色素瘤中，CD8^+^ TIL的丰度可预测对PD-1抑制剂的反应^[39]^。随着免疫治疗研究的深入，科学家们对免疫微环境的关注逐步提高，而TIL作为抗击肿瘤的主力军，其重要性不言而喻，但是目前关于TIL在SCLC中的研究主要局限在小样本的回顾性研究中，以后尚需更多的样本或前瞻性研究进一步确证其预测价值。

### 2.2 PD-L1表达部位

目前对于PD-L1的评估和样本来源也有所争议。一项关于Pembrolizumab用于ES-SCLC维持治疗的Ⅱ期临床研究^[40]^显示，在SCLC中，PD-L1在间质中的表达远高于肿瘤细胞内。如在一项纳入了104例Ⅰ期-Ⅲ期SCLC的研究^[41]^中，肿瘤细胞和TIL上PD-L1表达阳性的比例分别为25%和40%。Rivalland等^[42]^的另一项研究中这一比例则相差更多，肿瘤细胞和TIL的表达阳性率分别为18%和67%。Zimmermann等^[43]^研究发现，在多个瘤种中，TIL高表达PD-L1的肿瘤对Atezolizumab有反应。此外，综合比例得分（composite proportion score, CPS）同时结合了肿瘤细胞及免疫细胞的PD-L1表达情况，呈现出与应答更佳的相关性。

### 2.3 其他

调节性T（regulatory T, Treg）细胞可以抑制其他T细胞的活化、增殖和效应。过多的Treg细胞可能导致免疫监视功能的失活从而促进肿瘤进展。研究报道在SCLC肿瘤活检中发现了FOXP3^+^ T细胞，并且比例较高的患者生存预后较差^[44]^，阻断与Treg细胞诱导相关的IL-15可减弱其抑制作用。髓系来源的抑制性细胞（myeloid-derived suppressor cell, MDSC）是具有免疫抑制功能的细胞群，可以通过多种途径介导免疫逃逸和免疫耐受。在一项纳入了41例局限期SCLC患者的临床试验中，研究了MDSC对p53肿瘤疫苗反应的影响，试验中应用了全反式维甲酸使MDSC数量下降2倍以上，实验组中有41.7%对疫苗产生了免疫应答，而这一比例在对照组中仅为20%^[45]^。另一项回顾性研究^[46]^则探索了SCLC患者中MDSC的数量、密度和临床预后的关系，这也是首个证实MDSC与SCLC患者的临床结局呈负相关的临床试验。综上，Treg细胞及MDSC均被证实对免疫治疗产生影响，由此可见肿瘤微环境的重要性。肿瘤微环境是正向与负向多重因素相互交融、彼此作用的场所，相关的研究应该综合、全面地反映其真实情况。故多色免疫组化等检测手段可以进一步应用到该领域，全面且深入探索其预测价值。

## 3 循环标志物的检测

### 3.1 循环肿瘤细胞（circulating tumor cell, CTC）

CTC指自发或因诊疗操作脱离原发灶或者转移灶的具有高活力、高转移潜能，进入外周血液循环的一类肿瘤细胞。CTCs是肿瘤转移的主要途径，也是肿瘤筛查、诊断、预后、疗效评估的重要标记物。有研究^[47]^表明，当影像学可检测的结节性病变癌症病灶直径 > 1 cm时，肺癌患者外周血液中已经可以检测到CTCs。既往研究^[48, 49]^证实CTC在接受放、化疗的SCLC患者中具有良好的预测价值，但是目前尚无CTC在接受免疫治疗的SCLC患者的疗效预测研究。

目前CTC在免疫治疗的预测价值主要集中在其他瘤种的相关研究。在一项纳入了35例接受PD-1抑制剂IBI308治疗的实体瘤患者的临床试验^[50]^中，研究发现CTC数目的动态变化可以预测免疫治疗疗效，在11例临床获益的患者中，有73%的患者CTC数目减少，而在进展的患中有89%的患者CTC数量升高。此外，研究还进一步对CTC上PD-L1表达水平与免疫治疗疗效预测之间的关系展开研究，研究者根据CTC的PD-L1表达情况分为阴性、低、中、高四组，高表达组的疾病控制率（58%）远高于其他组（14%）。另一项意大利的研究^[51]^则纳入了24例接受Nivolumab治疗的Ⅳ期NSCLC患者，在基线、治疗3个月及6个月分别对表达PD-L1的CTC进行评估，该研究发现，在接受治疗6个月后，CTC上PD-L1表达转阴的患者具有较好的临床获益，而持续阳性的患者预后则较差，提示CTC持续表达PD-L1可能促进肿瘤的免疫逃逸。与组织标本相比而言，外周血标本更易获得，且对患者的创伤更小，因此具有一定的发展前景，但是由于CTCs的检测方法要求较高，且目前尚无统一的截断值，所以CTCs在免疫治疗中的研究仍需要进一步探索。

### 3.2 循环肿瘤DNA（circulating cell-free tumor DNA, ctDNA）

ctDNA是指由肿瘤脱落入循环系统的DNA片段。由于SCLC的快速进展并随着早期血行传播的高转移性，因此在SCLC患者中极易检测到ctDNA。一项研究^[52]^对SCLC患者的组织标本、外周血标本应用高通量目标基因测序技术进行深度测序。结果显示94%肿瘤DNA来源的体细胞突变都可以在ctDNA中检测出。同时也发现，治疗前ctDNA主克隆丰度较高的患者相比低于平均值的患者而言预后较差（PFS：5.3个月*vs* 10.0个月，*P*=0.002）。该研究进一步地对比了不同时期的ctDNA和影像学检查结果，ctDNA水平的动态变化与影像学上病灶大小密切相关。由于ctDNA的时效性及获得性均优于组织标本，因此也为SCLC预后提供了新的生物标记物的选择。

### 3.3 中性粒细胞/淋巴细胞比值（neutrophil-to-lymphocyte ratio, NLR）

NLR是反映抗肿瘤炎性的标记物，由于其便捷性及覆盖广等特点，在近年引起了学者们的关注。一项纳入了20项研究^[53]^共21个结果包括5, 141患者的*meta*分析探索了NLR对于SCLC的生存预测作用，结果显示治疗前高NLR与预后呈负相关（PFS: HR=1.55, 95%CI: 1.27-1.88, *P* < 0.000, 1; *I*^2^=0%; OS: HR=1.40, 95%CI: 1.26-1.55, *P* < 0.000, 01; *I*^2^=64%）。在今年癌症免疫治疗学会年会上，俄亥俄州立大学的一项研究^[54]^入组了78例晚期NSCLC免疫治疗的患者，结果显示治疗前NLR≥5的患者中位OS为10.7个月，而NLR < 5的患者则为46.1个月。研究者进一步观察了基线NLR与PD-L1表达的相关性，基线NLR < 5和NLR≥5患者的PD-L1肿瘤比例评分中位数分别为60%和20%。综上，NLR对免疫治疗的预测价值已初露锋芒，但是目前的研究也多集中在回顾性分析，所以后期前瞻性研究可以进一步证实其预测价值。

## 4 小结与展望

PD-1/PD-L1等免疫抑制剂给SCLC患者带来新的希望。然而，不论是在IMpower133亦或是CASPIAN研究，均能发现在其生存曲线的前6个月，治疗组与安慰机组非常接近，甚至有交叉的趋势，这便提示我们筛选受益群体的重要性。SCLC免疫治疗的疗效预测指标探索仍在初始阶段。PD-L1表达水平在SCLC中较低的阳性率可能会影响它对SCLC患者免疫治疗的预测价值。TMB的预测价值主要体现在免疫单药方面，然而免疫联合治疗是ES-SCLC患者的主要一线选择，所以未来的研究应该进一步优化TMB的算法并使之更好地区分免疫联合治疗的优势人群。其他的预测指标，如DDR、TIL等都被认为是非常有前景的预测标志物，值得进一步地研究验证与考究。虽然目前对于循环标志物在SCLC中应用的研究尚少，但是鉴于其他瘤种的报道及其便利性、技术的可操作性强，循环标志物也具有一定的研究前景。SCLC以免疫豁免型为主，对免疫单药治疗反应不佳，免疫联合治疗是SCLC的主要治疗手段，单一的疗效预测指标也许并不是最优解。通过动态组合策略或建立预测模型，综合应用多个平台一起实现对免疫治疗最佳疗效的预测也许将是未来前进的方向。综上，寻找最佳的免疫治疗疗效的预测标记物任重而道远。相信在不远的将来，SCLC患者可在精准的预测“开路”下，得到更高效的免疫治疗。
